# Expert consensus on diagnostic guidelines for pediatric inflammatory bowel disease in Japan

**DOI:** 10.1007/s00535-025-02271-7

**Published:** 2025-07-02

**Authors:** Takahiro Kudo, Katsuhiro Arai, Itaru Iwama, Shin-ichiro Hagiwara, Takashi Ishige, Koji Yokoyama, Fumihiko Kakuta, Keisuke Jimbo, Hiroki Kondou, Yugo Takaki, Shingo Kurasawa, Hiroki Fujikawa, Yuhki Koike, Fumihito Hirai, Shinya Ashizuka, Kenji Watanabe, Toshiaki Shimizu, Tadakazu Hisamatsu

**Affiliations:** 1 Department of Pediatrics, Juntendo University Faculty of Medicine, 2-1-1 Hongo, Bunkyo-ku, Tokyo, 113-8421 Japan; 2 Division of Gastroenterology, Center for Pediatric Inflammatory Bowel Disease, National Center for Child Health and Development, Tokyo, Japan; 3 Division of Gastroenterology and Hepatology, Saitama Children’s Medical Center, Saitama, Japan; 4 Department of Pediatric Gastroenterology, Nutrition and Endocrinology, Osaka Women’s and Children’s Hospital, Osaka, Japan; 5 Department of Pediatrics, Gunma University Graduate School of Medicine, Gunma, Japan; 6 Department of Pediatrics, Jichi Medical University School of Medicine, Tochigi, Japan; 7 Department of Gastroenterology and Hepatology, Miyagi Children’s Hospital, Sendai, Japan; 8 Department of Pediatrics, Kindai University Nara Hospital, Nara, Japan; 9 Department of Pediatric Gastroenterology and Hepatology, Japanese Red Cross Kumamoto Hospital, Kumamoto, Japan; 10 Department of Pediatrics, Shinshu University School of Medicine, Matsumoto, Japan; 11 Department of Pediatrics, Hiroshima University Graduate School of Biomedical and Health Sciences, Hiroshima, Japan; 12 Department of Gastrointestinal and Pediatric Surgery, Mie University Graduate School of Medicine, Mie, Japan; 13 Department of Gastroenterology, Faculty of Medicine, Fukuoka University, Fukuoka, Japan; 14 Department of Internal Medicine for Inflammatory Bowel Disease, University of Toyama, Toyama, Japan; 15 Department of Gastroenterology and Hepatology, Kyorin University School of Medicine, Tokyo, Japan

**Keywords:** Children, Crohn’s disease, Inflammatory bowel disease, Pediatric, Ulcerative colitis

## Abstract

**Background:**

Inflammatory bowel disease (IBD) can occur at any age. In pediatric patients, the disease may present with a broader range of symptoms and more severe course than in adults, due to ongoing growth and development. Therefore, pediatric IBD often exhibits an atypical clinical course and laboratory findings. It is essential to recognize differences in disease presentation, differential diagnoses, and evaluation strategies specific to children. The revised Porto criteria, proposed by the European Society for Paediatric Gastroenterology, Hepatology, and Nutrition (ESPGHAN) in 2014, are widely used globally, including in Japan, for the diagnosis of pediatric IBD.

**Purpose:**

Despite the widespread use of these criteria, no formal diagnostic guidelines for pediatric IBD have been developed in Japan. We aimed to support future guideline development by summarizing important diagnostic considerations and clinical practices for pediatric IBD in Japan.

**Methods:**

This review was developed based on relevant international diagnostic guidelines and the expert opinions of Japanese pediatric gastroenterologists. It outlines key clinical and laboratory evaluations, as well as current treatment and follow-up approaches.

**Results:**

We summarized recommended diagnostic tests and clinical points that require special attention in children with suspected IBD. The article reflects both global standards and domestic clinical experience.

**Conclusion:**

Although this article does not provide formal diagnostic criteria or assess evidence levels, it offers accurate and practical information to guide physicians and patients in the diagnosis and management of pediatric IBD in Japan.

## Introduction

**Fig. 1:**
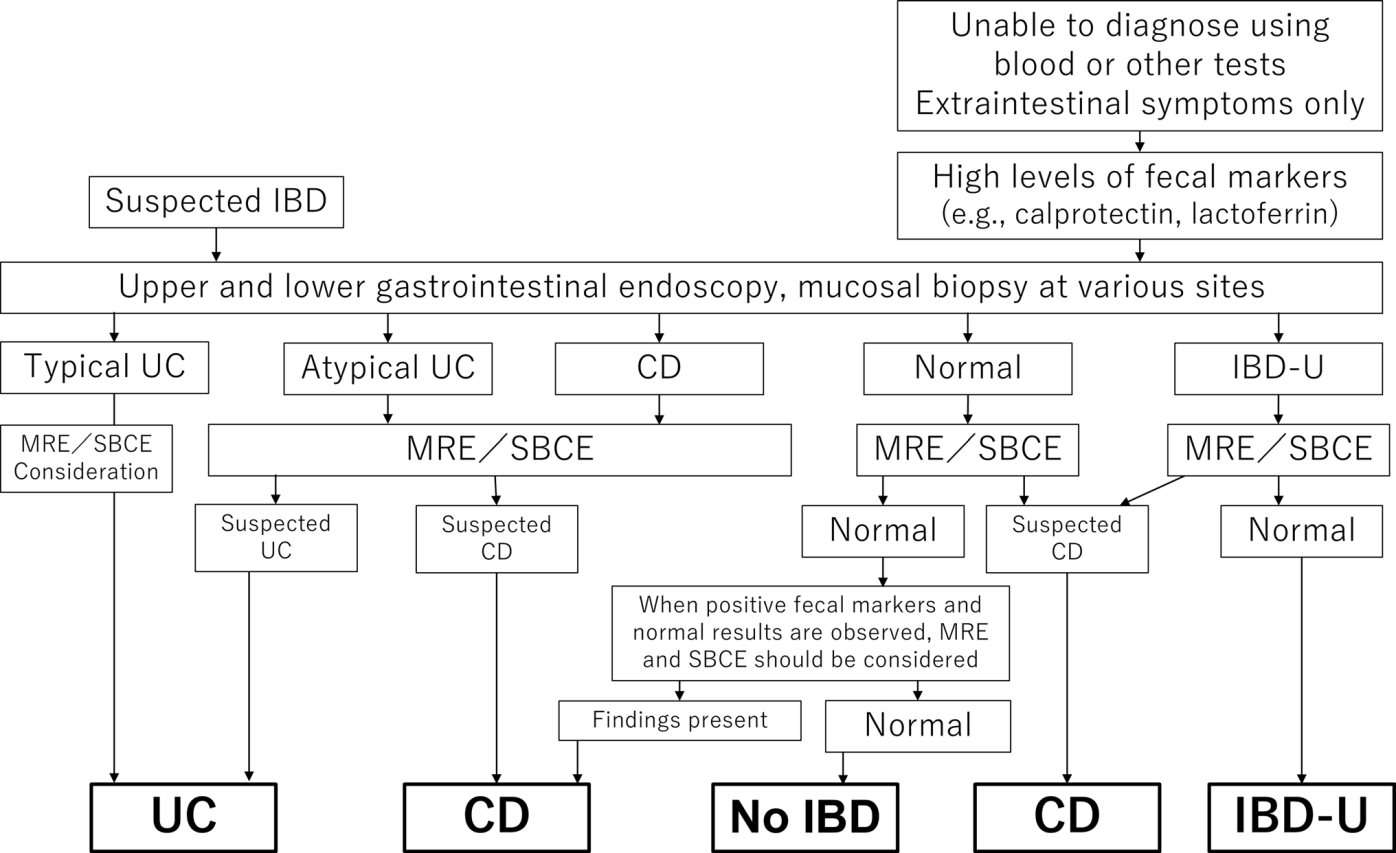
Diagnostic flowchart of pediatric inflammatory bowel disease (IBD) proposed by the European Society for Paediatric Gastroenterology, Hepatology, and Nutrition (ESPGHAN). Because of the risk of capsule retention in children, small bowel patency should be evaluated using a patency capsule before attempting small bowel capsule endoscopy (SBCE). *BAE* balloon enteroscopy, *CD* Crohn’s disease, *IBD-U* inflammatory bowel disease unclassified, *MRE* magnetic resonance enterography, *UC* ulcerative colitis

Inflammatory bowel disease (IBD) is characterized by chronic inflammation of the gastrointestinal tract of unknown cause and gastrointestinal symptoms, such as abdominal pain, diarrhea, and bloody stools with repeated flare-ups and relapses [1]. IBD is typically classified as either ulcerative colitis (UC) or Crohn’s disease (CD). The diagnostic criteria for IBD in Japan have been developed by the Ministry of Health, Labour, and Welfare Research Group and applied to pediatric IBD. IBD is commonly diagnosed between approximately 20 and 40 years of age; however, 12.1% of all UC cases and 23.7% of all CD cases are diagnosed before the age of 20 [2], indicating the high severity and wide range of the clinical manifestations of IBD. A Japanese pediatric IBD registry study indicated that the extent of IBD in Japan is not significantly different from that in Europe in terms of the prevalence of UC, but that CD is more extensive in Japan, with more patients experiencing anorectal involvement [3]. Because many patients exhibit atypical clinical symptoms and laboratory findings, IBD unclassified (IBD-U), which is a typical form of IBD that does not meet the diagnostic criteria for UC or CD, may be diagnosed [4, 5]. IBD-U is diagnosed using the diagnostic procedures for UC and CD in Japan, and its progression is carefully followed. The frequency of IBD-U is higher among children than among adults because of the high prevalence of atypical cases and lack of adequate laboratory tests [4]. Additionally, compared to adult-onset IBD, pediatric IBD and very early-onset IBD (occuring before age 6) may be associated with underlying factors, such as immunodeficiency and single-gene disorders, and are more strongly related to genetic factors; therefore, genetic analyses are necessary for some cases. Furthermore, the possibility of stunted growth must be considered when determining the optimal treatment for pediatric patients with IBD. Therefore, understanding that pediatric IBD represents a distinct condition with different courses and clinical laboratory findings that should be differentiated from adult IBD is essential. The worldwide diagnostic criteria used for pediatric IBD are the revised Porto criteria (Fig. 1), which were proposed by the European Society for Paediatric Gastroenterology, Hepatology, and Nutrition (ESPGHAN) [6]. In case the patients present with symptoms suggestive of IBD, or have stool analysis results indicating abnormally high levels of calprotectin or lactoferrin, particularly in the presence of laboratory test findings or extraintestinal symptoms suggestive of IBD, upper gastrointestinal endoscopy, lower gastrointestinal endoscopy, including the terminal ileum, and histopathological examination with a mucosal biopsy are recommended. For such cases, small bowel capsule endoscopy (SBCE) or magnetic resonance enterography (MRE) should be performed to evaluate the small intestinal lesions. The findings of SBCE and MRE should be carefully evaluated when a disease other than typical UC is suspected. Additionally, a diagnosis of UC, CD, IBD-U, or non-IBD should be made according to these findings. Owing to the risk of capsule retention in children, a small bowel patency evaluation using a patency capsule should be performed prior to SBCE. However, depending on the course of clinical symptoms, gastrointestinal endoscopy should be repeatedly performed over time to consider any changes in the diagnosis. Very early-onset IBD (VEO-IBD), defined as onset before the age of 6, often presents with atypical features and is frequently associated with underlying monogenic disorders or primary immunodeficiencies. Although this consensus primarily addresses general pediatric IBD diagnosis, future guidelines should incorporate genetic screening strategies and diagnostic algorithms tailored to this unique subset of patients.

### Ulcerative colitis

The main symptoms of UC are diarrhea, bloody stools, and abdominal pain. These symptoms are frequently observed in children. For children with chronic persistent symptoms, lower gastrointestinal endoscopy, including the terminal ileum, and histopathological examinations should be performed. In case diarrhea and abdominal pain are present and bleeding symptoms are not evident, blood tests should be performed and stool biomarkers should be used to determine whether lower gastrointestinal endoscopy is indicated. Patients with pediatric UC often have a history of antimicrobial use and international travel. Compared to adults, children are more likely to have a family history of IBD; therefore, the presence of IBD in the extended family history should be confirmed [6]. Endoscopy for children comprises several challenges, including the selection of appropriate endoscopic equipment based on the patient’s body size, determination of safe and reliable levels of anesthesia, as well as the use of sedation during examinations, and management during and after examinations, with a particular focus on airway management. Additionally, few physicians and facilities in Japan specialize in pediatric gastrointestinal diseases, and some facilities are not equipped to perform endoscopic examinations. However, upper and lower gastrointestinal endoscopies, including the terminal ileum, can be performed for children of all ages and sizes using the available endoscopes. These examinations can be performed safely if the patient is managed by a skilled pediatrician or, in some cases, an anesthesiologist during and after the procedures. Typical UC can be diagnosed based on the results of lower gastrointestinal endoscopy, including the terminal ileum, and histopathology. However, for younger patients, those with upper gastrointestinal symptoms, and those with atypical lower gastrointestinal endoscopy and histopathology results, small bowel lesions should be examined using small bowel endoscopy and imaging tests, as well as upper gastrointestinal endoscopy. Stool cultures to test for the presence of bacteria and parasites should be performed to determine the possibility of infectious enteritis. Although performing diagnostic tests for pediatric patients proves challenging, an accurate diagnosis is necessary to determine effective treatment. Additionally, as many patients with pediatric UC have more severe disease or extensive lesions compared to those of adults, and because the prognosis of UC can be determined by the disease severity at the time of the diagnosis, prompt diagnoses and treatment are critical [7, 8].

### Crohn’s disease

Because CD can involve the entire gastrointestinal tract from the mouth to the anus, a complete gastrointestinal evaluation is necessary to determine the diagnosis. Although the main symptoms are abdominal pain and diarrhea, gastrointestinal symptoms are minor in some cases, and recurrent fever, extraintestinal complications, and growth restriction are more prominent. Therefore, the process necessary to determine the diagnosis is often time-consuming. When the symptoms, physical examination results, and blood test results suggest CD, a full gastrointestinal examination should be planned. The diagnosis of CD in adults and children is determined based on endoscopic findings [6]. Upper and lower gastrointestinal endoscopies, including the terminal ileum, can be performed for children of all ages and weights using the available endoscopes. Capsule and balloon endoscopes can be used for young patients, with some restrictions, to search for lesions in the small intestine. However, when endoscopy is difficult, small bowel contrast, magnetic resonance imaging, or computed tomography can be used to search for small bowel lesions. The complication rate of small bowel lesions is higher for pediatric CD than for adult CD [8]. A thorough search for small bowel lesions can result in an early diagnosis of CD and avoid an unnecessary diagnosis of IBD-U. Thorough examinations are necessary to make an accurate diagnosis, locate lesions, select effective treatment, and determine the prognosis.

### Inflammatory bowel disease unclassified

IBD-U is defined as inflammation located mainly in the colon; however, the diagnosis of IBD is definitive. IBD-U is diagnosed when the results of a thorough examination of the entire gastrointestinal tract do not indicate UC or CD [6]. The ratio of IBD-U cases to total IBD cases is higher for children than for adults, and many cases are subsequently rediagnosed as UC or CD [9, 10]. However, the diagnosis of IBD-U is retained for many cases [11]; therefore, the treatment of IBD-U during its natural history and the pathophysiology of IBD are paramount.

## Methods

A grant from the Ministry of Health, Labour and Welfare (MHLW) for research on intractable diseases was obtained to support the establishment of a task force to build consensus among experts on matters necessary to diagnose patients with childhood-onset inflammatory bowel disease (IBD). First, a steering committee consisting of 14 pediatric gastroenterologists and 4 gastroenterologists was organized. The committee was tasked with developing a questionnaire for a systematic literature search. The literature review team conducted a systematic literature search using PubMed and the Medical Journal (www.jamas.or.jp). Gray literature, including government alerts and National Institute of Infectious Diseases statistics, was also included. The review team consisted of 18 physicians, including pediatric gastroenterologists and gastroenterologists, and was divided into nine clinical questions. Expert committees on the diagnosis of pediatric IBD, endoscopy, pathology, small bowel examination, abdominal ultrasound, biomarker utility, growth disturbances, classification, and considerations in severity jointly discussed and drafted comments. Draft comments were discussed with the Steering Committee members divided into sections and a statement was prepared. A web conference was then held with the participation of all committee members to reach a consensus among them. After some minor revisions were made based on the discussions at the meeting, the consensus of all committee members was confirmed electronically. The manuscript was made available to all members of the Intractable Disease Research Grant for comment prior to submission. However, this consensus building process did not determine the strength of the recommendation.

## Diagnostic guidelines for pediatric inflammatory bowel disease in Japan: clinical questions

Clinical Question (CQ) 1: How is the diagnosis of pediatric inflammatory bowel disease determined?

CQ2: Are upper and lower gastrointestinal endoscopies useful for diagnosing pediatric IBD?

CQ3: Is histopathology useful for diagnosing pediatric inflammatory bowel disease?

CQ4: Are small bowel capsule endoscopy, balloon enteroscopy, and magnetic resonance enterography useful for diagnosing pediatric inflammatory bowel disease?

CQ5: Is abdominal ultrasonography useful for diagnosing pediatric inflammatory bowel disease?

CQ6: Are biomarkers useful for diagnosing pediatric inflammatory bowel disease?

CQ7: What are the causes of growth restriction with pediatric inflammatory bowel disease?

CQ8: Which classification is recommended for pediatric inflammatory bowel disease?

CQ9: How is the severity of pediatric inflammatory bowel disease classified?

### CQ1: How is the diagnosis of pediatric inflammatory bowel disease determined?

#### Statement

- Pediatric IBD is diagnosed according to the criteria developed by the Ministry of Health, Labour, and Welfare.
- It is important to exclude infectious enteritis before determining the diagnosis.
- As few atypical cases exist that do not meet the diagnostic criteria of childhood-onset cases, upper and lower gastrointestinal endoscopies, including the terminal ileum, should be performed for all cases of suspected IBD. For cases other than typical UC, SBCE, MRE, and other imaging evaluations of small bowel lesions are recommended.
- The diagnosis of IBD-U is based on pediatric IBD classes.

#### Commentary

The diagnostic criteria for UC and CD developed by the Ministry of Health, Labour, and Welfare are used to diagnose pediatric IBD (Table 1) [1]. Therefore, the diagnostic procedures and criteria for children follow those for adults. An accurate diagnosis of IBD requires a review of the medical history, as well as results of physical examination, blood test, upper and lower gastrointestinal endoscopies, including the terminal ileum, histopathology, and imaging studies of the small intestine. Compared to the European and United States diagnostic criteria, the Japanese diagnostic criteria for IBD emphasized more on imaging findings. Before determining the diagnosis, distinguishing the gastrointestinal infections is essential by performing a stool culture and parasitological examination. The differential diagnoses of pediatric IBD are listed in Table 2 [12–14].

**Table 1:** Diagnostic criteria for ulcerative colitis

A. Clinical manifestations: persistent or recurrent mucous-containing or bloody stools or a history of mucous-containing or bloody stools
B. (1) Endoscopic examination: (i) the mucosa is diffusely affected and coarse or finely granular, vascular translucency is absent, and lesions are fragile and easily hemorrhagic (hemorrhage on contact), with mucous and purulent secretions; (ii) multiple erosions, ulcers, or pseudopolyposis are present; and (iii) lesions are contiguous to the rectum (2) Intestinal radiography: (i) diffuse changes of the mucosal surface in the form of coarse or fine granules; (ii) multiple erosions and ulcers; or (iii) pseudopolyposis. Other findings include loss of the haustra (lead pipe appearance) and narrowing or shortening of the intestinal tract
C. Histopathology: during the active stage, diffuse inflammatory cell infiltration of the entire mucosal layer, crypt abscesses, and severe goblet cell depletion are observed. All of these findings are nonspecific and should be comprehensively evaluated. During remission, an abnormal glandular arrangement (meandering and branching) and atrophy remain. These changes are usually observed continuously from the rectum to the mouth
Examples of the confirmed diagnosis: 1. Cases that satisfy (1) or (2) of (B) or (C) in addition to (A) 2. Cases that satisfy (1) or (2) of (B) and (C) more than once during the course of disease 3. Cases with gross and histologic findings characteristic of disease observed during surgical resection or autopsy
Note 1: A confirmed case is defined as a case for which the following diseases should be excluded: infectious enteritis such as bacterial dysentery, *Clostridioides difficile* enteritis, amoebic colitis, *Salmonella* enteritis, *Campylobacter* enteritis, colonic tuberculosis, and *Chlamydia* enteritis, and other diseases, such as Crohn’s disease, radiation colitis, drug-induced colitis, lymphoid follicular hyperplasia, ischemic colitis, and intestinal Behçet’s disease
Note 2: If the diagnosis is not definite because of mild findings, then the diagnosis should be treated as uncertain
Note 3: When differentiating Crohn’s disease from ulcerative colitis proves difficult, follow-up should be performed. If a definitive diagnosis cannot be made based on the clinical picture, including endoscopic and biopsy findings, then the case should be classified as inflammatory bowel disease unclassified. If a definitive diagnosis cannot be made after a histopathological examination of the resected specimen, then the case should be classified as indeterminate colitis. During follow-up, more characteristic findings of either disease may emerge
Note 4: Familial Mediterranean fever may present with colorectal lesions, thus resembling ulcerative colitis, and may require differentiation based on the clinical course and other factors

Adapted from [1], with partial modification

**Table 2:** Gastrointestinal diseases requiring differentiation from pediatric inflammatory bowel disease

Infectious enteritis Viral enteritis (including cytomegalovirus), bacterial enteritis (*Salmonella*, *Campylobacter*, Yersinia, and others), intestinal tuberculosis, *Clostridioides difficile*
Eosinophilic gastrointestinal diseases
Vascular lesions IgA vasculitis, ANCA-associated vasculitis, Takayasu’s disease, intestinal Behçet’s disease, ischemic enteritis
Primary immunodeficiency Chronic granulomatosis, interleukin-10 receptor deficiency, A20 haploinsufficiency, X-linked lymphoproliferative syndrome type 2, Wiskott-Aldrich syndrome, IPEX syndrome, and others
*SLCO2A1*-associated small bowel disease (chronic enteropathy associated with the *SLCO2A1* gene)
Celiac disease
Drug-induced enteritis Induced by nonsteroidal anti-inflammatory drugs and others

*ANCA* anti-neutrophilic cytoplasmic autoantibody, *Ig* immunoglobulin A, *IL* interleukin, *IPEX* immunodysregulation, polyendocrinopathy, enteropathy, X-linked

Atypical cases of UC have been observed more often in adults than in children [15]. The revised Porto criteria proposed by ESPGHAN are currently used worldwide as the diagnostic criteria for pediatric IBD, and they recommend upper and lower gastrointestinal endoscopies including the terminal ileum, and a histological examination, including a biopsy of the gastrointestinal mucosa for all pediatric patients with suspected IBD [6]. Atypical lower gastrointestinal endoscopic findings of UC include the following: (1) rectal sparing (inflammation of the rectal mucosa that is grossly mild or appears completely normal compared to the mucosa on the oral side of the rectum); (2) short duration (atypical findings that do not meet the diagnostic criteria because of short disease durations); (3) cecal patch (left-sided colitis with localized inflammation near the appendiceal orifice); (4) upper gastrointestinal tract involvement (erosions and nonlongitudinal ulcers in the upper gastrointestinal tract seen with ulcerative colitis); and (5) acute severe colitis with backwash ileitis (retrograde ileitis comprises nonerosive erythema, edema, and other inflammation in the terminal ileum that is continuous from the cecum). For cases involving symptoms other than those of typical UC, evaluations including imaging, such as SBCE or MRE, of small intestinal lesions are recommended. The calprotectin stool test, serum anti-integrin αVβ6 antibody test, and immunological fecal occult blood test are useful for diagnosing UC [16, 17]. The diagnostic ability of serum anti-integrin αVβ6 antibody for UC is considerably good for both adult (sensitivity, 92.0%; specificity, 94.8%) [16] and pediatric cases (sensitivity, 94.7%; specificity, 81.3%) [17].

Pediatric CD can be diagnosed according to the criteria developed by the Ministry of Health, Labor, and Welfare (Table 3) [1]. Similar to pediatric UC, atypical cases of CD that do not meet the diagnostic criteria have been recognized. Although the typical features of pediatric CD are aphthous or longitudinal ulcers in the ileum or colon, they can occur throughout the gastrointestinal tract, and their histology is characterized by chronic inflammation with or without nondesmoplastic epithelioid cell granulomas [6]. In the absence of these findings, the possibility of UC or IBD-U should be considered. IBD during infancy and colonic CD may be difficult to differentiate from UC or IBD-U [18, 19]. Limbergen et al. reported that approximately 5% of pediatic patients with CD presented with only oral or anal lesions, with no abnormalities in the gastrointestinal mucosa; of these patients, 70% experienced gastrointestinal lesions during ≥ 4 years of follow-up [20]. Therefore, careful long-term follow-up is important even when patients present with only persistent anorectal lesions.

**Table 3:** Diagnostic criteria for Crohn’s disease

(1) Primary findings A. Longitudinal ulcer (Note 1) B. Cobblestone appearance C. Nondysbutyroid epithelioid cell granuloma (Note 2)
(2) Secondary findings a. Irregular or round ulcers or aphthae over a large area of the gastrointestinal tract (note 3) b. Characteristic anal lesions (Note 4) c. Characteristic gastric or duodenal lesions (Note 5)
Examples of a confirmed diagnosis: 1. Primary finding (A) or (B) (Note 6) 2. Primary (C) and secondary findings (a) or (b) 3. Secondary findings (a), (b), and (c)
Example of an uncertain diagnosis: 1. Primary finding (C) and secondary finding (c) 2. Primary finding (A) or (B) but the case cannot be differentiated from ulcerative colitis, intestinal Behçet’s disease, simple ulcers, or ischemic bowel lesions 3. Primary finding (C) (Note 7) 4. One or two secondary findings
Note 1: Longitudinal ulcers are observed along the long axis of the intestinal tract and have a predilection for attachment on the mesenteric side in the small intestine. These ulcers are typically 4 to 5 cm or longer; however, this length is not a required feature
Note 2: Serial sectioning improves the diagnostic yield. A pathologist familiar with the gastrointestinal tract should make the diagnosis
Note 3: Extensive involvement of the gastrointestinal tract indicates that the lesion is anatomically distributed over multiple organs, such as the upper gastrointestinal tract (esophagus, stomach, duodenum), small intestine, and large intestine. Although the lesions are typically longitudinal, they may not be in some cases. The lesion must persist for at least 3 months. Capsule endoscopic findings may indicate multiple rings on the Kerckring folds in the duodenum and small intestine. Intestinal tuberculosis, intestinal Behçet’s disease, simple ulcers, ulcers caused by nonsteroidal anti-inflammatory drugs, and infectious enteritis should be ruled out
Note 4: Characteristic anal lesions include anal fissures, cavitating ulcers, hemorrhoids, perianal abscesses, and edematous dermatomes. A proctologist familiar with Crohn’s disease should make the diagnosis
Note 5: Characteristic gastric or duodenal lesions have a bamboo knot-like appearance and notch-like depression. The diagnosis should be made by a Crohn’s disease specialist
Note 6: If only a longitudinal ulcer is present, then ischemic bowel disease or ulcerative colitis should be ruled out. If only a cobblestone appearance is present, then ischemic bowel disease or type 4 colorectal cancer should be ruled out
Note 7: Inflammatory diseases with granulomas, such as intestinal tuberculosis, should be excluded

Adapted from [1], with partial modification

**Fig. 2:**
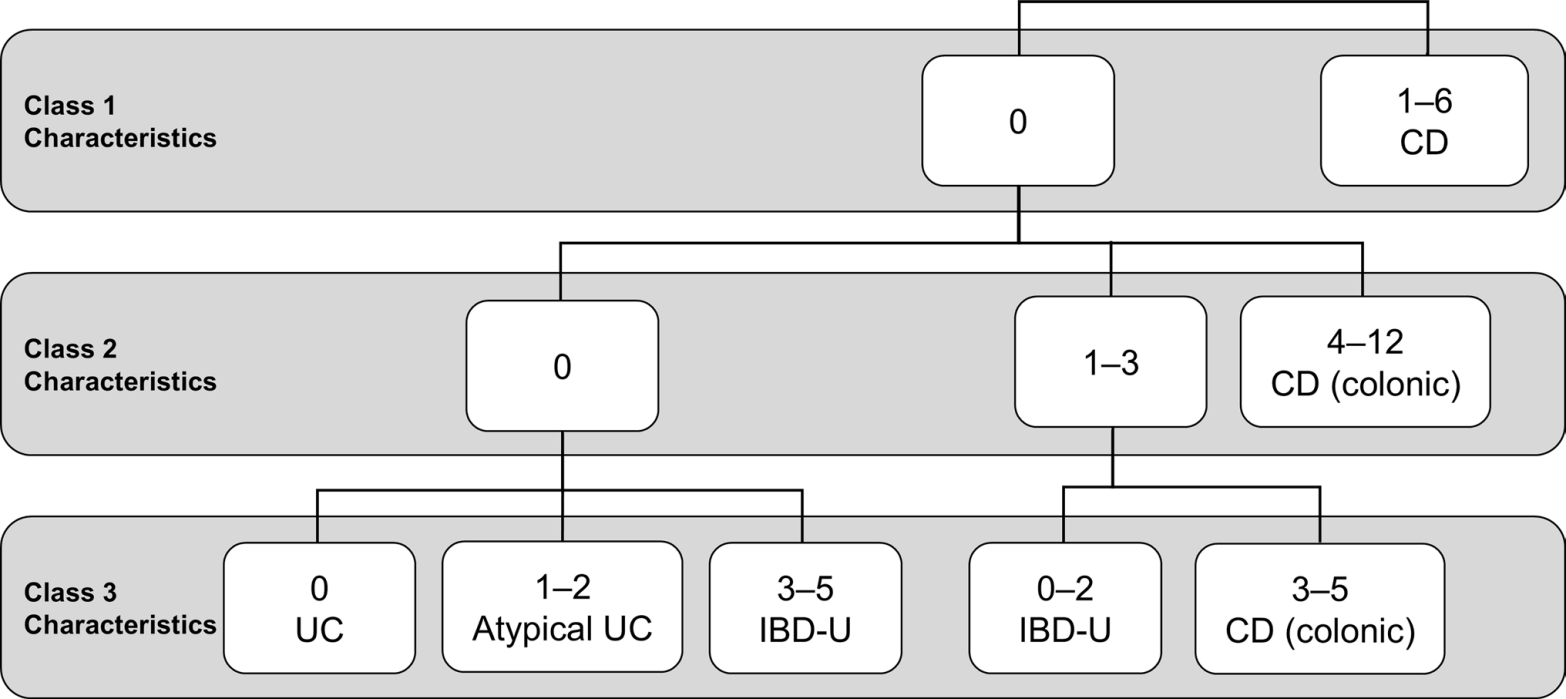
Algorithm of pediatric inflammatory bowel disease (IBD) classes used to determine the differential diagnosis of IBD subgroups. Adapted from [10], with partial modification. Upper row: number of applicable items among six features (no. 1–6 of class 1 of Table 4). Middle row: number of corresponding items among 12 features (no. 7–18 of class 2 of Table 4). Lower row: number of corresponding items among five features (no. 19–23 of class 3 of Table 4). *CD* Crohn’s disease, *IBD-U* inflammatory bowel disease unclassified, *UC* ulcerative colitis

IBD-U is defined as inflammation in the colon with a definite diagnosis of IBD but without a diagnosis of UC or CD after careful examination [21, 22]. The ESPGHAN Paediatric IBD Porto Group and the European Crohn’s and Colitis Organization (ECCO) have proposed pediatric IBD classes that can be used to separate pediatric IBD into five categories (typical UC cases, atypical UC cases, IBD-U, colonic CD, and CD) (Table 4) [6, 21–24]. Pediatric IBD cases are categorized into three classes based on the presence of 23 features of colitis that can be observed with CD: class 1, cases without any of these features of colitis (these should be diagnosed as CD); class 2, cases that rarely exhibit some of these features of colitis (< 5%; the frequency of the observation of these features with cases); and class 3, cases that occasionally exhibit some of these features of colitis (5–10%). Cases are weighted according to their class to standardize the diagnosis of pediatric IBD (Fig. 2). These diagnostic criteria facilitate the clear classification of the diagnosis of IBD-U. However, in actual clinical practice, most cases of IBD-U are treated as either UC or CD, reducing the need for strict classifications. A diagnosis of IBD-U may later shift to typical UC or CD during the clinical course, requiring careful follow-up.

**Table 4:** Diagnostic characteristics of untreated pediatric ulcerative colitis

	Characteristics
Class 1: Never found with UC	1. At least one granuloma in any part of the gastrointestinal tract that is distal to the ruptured crypt 2. One deep ulcer, cobblestone appearance, or stricture anywhere in the small intestine or upper digestive tract (but not in the stomach) 3. Fistula in the gastrointestinal tract or perianal region 4. Presence of a large perianal inflammatory dermoid 5. Imaging studies indicating thickening of the jejunum or ileum or capsule endoscopy indicating significant small bowel inflammation that is not backwash ileitis 6. Significant inflammation of the ileum not caused by backwash ileitis
Class 2: Rarely found (< 5%) with UC	7. Endoscopically and histologically normal skip lesions, but not rectal sparing or cecal patch 8. Endoscopically and histologically complete rectal sparing (normal rectal sparing comprises a histologic lesion in the rectum) 9. Histologic evidence of inflammation in the colonic mucosa despite endoscopic biopsy of a normal portion of the colon with evidence of surrounding inflammation 10. Severe growth restriction (growth rate standard deviation < − 2) that cannot be explained by other causes (e.g., long-term steroid use, growth hormone secretion failure) 11. Presence of generalized inflammation of the colon in the absence of severe colitis 12. Presence of small, shallow ulcers (including aphthous ulcers) in the small intestine, duodenum, or esophagus (excluding the stomach and colon) that cannot be explained by other causes (*Helicobacter pylori* infection, NSAIDs) 13. Multiple (> 5) small, shallow ulcers (including aphthous ulcers) in the stomach or colon with normal background mucosa that cannot be explained by other causes (*H. pylori* infection, NSAIDs) 14. Backwash ileitis despite markedly mild inflammation in the cecum 15. Anti-*Saccharomyces cerevisiae* antibody-positive and perinuclear anti-neutrophil cytoplasmic antibody-negative findings 16. Inflammation of the colonic mucosa is more intense on the oral side than on the anorectal side (except for rectal sparing) 17. Severe scalloping of the stomach or duodenum that cannot be explained by other causes (e.g., *H. pylori* infection) 18. At least one deep ulcer or severe cobblestone appearance in the stomach that cannot be explained by other causes (*H. pylori* infection, NSAIDs)
Class 3: Occasionally found with UC (5–10%)	19. Histopathology reveals focal chronic duodenitis 20. Histopathology reveals localized active colitis in multiple specimens 21. Multiple (< 5) aphthous ulcers in the colon or stomach 22. Nonbloody diarrhea 23. Histopathology of the stomach indicates focal enhanced gastritis (gastritis forming a focal inflammatory cell infiltrate)

Adapted from [13], with partial modification

*NSAIDs* nonsteroidal anti-inflammatory drugs, *UC* ulcerative colitis

### CQ2: Are upper and lower gastrointestinal endoscopies useful for diagnosing pediatric inflammatory bowel disease?

#### Statement

- Upper gastrointestinal endoscopy is recommended for diagnosing pediatric IBD.
- Lower gastrointestinal endoscopy, including the terminal ileum is recommended for diagnosing pediatric IBD.
- For upper gastrointestinal endoscopy, biopsy specimens should be obtained from the esophagus, stomach, and duodenum, regardless of the presence or absence of symptoms or findings.
- For lower gastrointestinal endoscopy, including the terminal ileum, biopsy specimens should be obtained from each region.
- For cases of severe inflammation, observation may be limited to the sigmoid colon during the acute phase because of safety reasons; however, lower gastrointestinal endoscopy, including the terminal ileum should be performed after the acute phase ends.
- If the initial endoscopy does not yield a diagnosis, then a repeat examination should be considered.

#### Commentary

Gastrointestinal endoscopy is required to diagnose IBD. The ESPGHAN revised Porto criteria include a diagnostic index for pediatric IBD and advocate a comprehensive diagnosis based on a combination of upper gastrointestinal endoscopy results, including the clinical course, physical examination results, clinical laboratory data and histology results, results of the lower gastrointestinal endoscopy, including the terminal ileum, and results of the imaging evaluation of the small bowel [6, 25].

For children with suspected IBD, upper gastrointestinal endoscopy is recommended regardless of the presence or absence of upper gastrointestinal symptoms [6]. However, in Japan, no consensus exists regarding whether upper gastrointestinal endoscopy should be performed for all patients. Although the advantages and disadvantages of upper gastrointestinal endoscopy should be considered based on the situation of each institution (available examination systems, sedation methods), aggressive upper gastrointestinal endoscopy is recommended for atypical cases. Upper gastrointestinal endoscopy is particularly useful for diagnosing nonspecific colitis. According to the European-Israeli Registry Study (EUROKIDS) by ESPGHAN that included 1811 pediatric patients with IBD, 35% of patients with CD had abnormal upper gastrointestinal endoscopic findings; of these patients, 24% had findings characteristic of CD (aphthae, ulcers, cobblestone appearance, and stenosis) [26]. Additionally, a pediatric IBD registry study performed in Hungary revealed that upper gastrointestinal endoscopic findings (including one case of granuloma detected by pathological examination) were useful for determining the definitive diagnosis for 9% of patients (16/176 patients) with CD [27].

The end of the ileum was not observed in 20–25% of lower gastrointestinal endoscopies, including the terminal ileum, performed for suspected pediatric IBD cases [26]. A pediatric gastroenterologist or gastroenterologist with appropriate training should perform endoscopy for children with suspected IBD under the supervision of a physician with extensive pediatric IBD experience [25, 28, 29]. Additionally, age-appropriate pretreatment and sedation (general anesthesia or deep sedation) should be selected. The guidelines for pediatric gastrointestinal endoscopy jointly developed by the European Society of Gastrointestinal Endoscopy and ESPGHAN recommend that pediatric endoscopy be performed under general anesthesia. If the facility is not equipped to perform endoscopy under anesthesia, then endoscopy should be performed under deep sedation with adequate supervision [28, 29]. According to a study of 276 lower gastrointestinal endoscopies, including the terminal ileum, performed for children aged < 6 years (median age, 2.49 years; age range, 5 days to 5 years and 11 months) in Japan, 47% (130 procedures) were performed under general anesthesia, 45% (124 procedures) were performed under venous anesthesia, and 8% (22 procedures) were not performed under sedation, thereby highlighting that more procedures were performed under general anesthesia. The terminal ileum observation rate was higher under general anesthesia (44% vs. 85%) than that under venous anesthesia [30]. Although the sedation method of choice varies depending on the size of the facility and the system used, it is advisable to use a sedation method that ensures the safety and feasibility of endoscopy. In Japan, endoscopic examination for young children is sometimes performed under general anesthesia; a study of children under 6 years of age found that 47% were performed under general anesthesia. Therefore, while general anesthesia is generally considered necessary for children younger than 6 years of age in some institutions, they can undergo examinations under intravenous sedation if the facility has the capacity and the child cooperates [30]. The procedure must be performed under adequate supervision. A monitoring physician should be present when the procedure is performed under sedation. Careful monitoring of the patient’s condition post-examination is also necessary. Endoscopes are described in the American Society of Gastrointestinal Endoscopy Statement and the Japanese Guidelines for Pediatric Gastrointestinal Endoscopy 2017 [31]. The scope is often changed to one with a narrower diameter or a general-purpose scope for patients with a body weight of ≤ 10 kg (Table 5).

**Table 5:** Endoscope selection according to body weight

Weight	Upper gastrointestinal endoscopy	Lower gastrointestinal endoscopy
2.5–10 kg	A narrow upper gastrointestinal scope with an outer diameter ≤ 6 mm is recommended, especially for patients who weigh < 5 kg Consider a normal-diameter upper gastrointestinal scope if necessary (especially for therapeutic endoscopy)	A small- or normal-diameter upper gastrointestinal scope with an outer diameter ≤ 6 mm can be used for patients who weigh 5–12 kg
> 10 kg	A normal-diameter upper gastrointestinal scope can be used Therapeutic scopes can be selected for therapeutic endoscopy	A colonoscope with an outer diameter of approximately 11 mm or a typical colonoscope can be used

In nonurgent situations, upper and lower gastrointestinal endoscopies are basic procedures; however, lower gastrointestinal endoscopy including the terminal ileum is mandatory as rectal sigmoidoscopy and incomplete lower gastrointestinal endoscopy are not sufficient. During the acute phase of severe cases, it may be safe to limit observations to the sigmoid colon; however, in such cases, lower gastrointestinal endoscopy including the terminal ileum should also be performed in the remission stage after the acute phase [25]. Children with CD are more likely to have colorectal lesions than adults; therefore, sigmoidoscopy and partial colonoscopy should not be used to rule out CD [32]. While upper and lower gastrointestinal endoscopies, including terminal ileum observation, are recommended for all suspected pediatric IBD cases, it is acknowledged that such comprehensive evaluation may not be feasible in all healthcare settings, particularly in regional or low-resource areas. In such cases, initial evaluation with available modalities and timely referral to specialized centers should be considered to ensure accurate diagnosis and management.

The ESPGHAN and ECCO guidelines recommend that multiple biopsy samples (minimum of two per region) should be obtained from five regions (terminal ileum, cecum, transverse colon, sigmoid colon, and rectum) during lower gastrointestinal endoscopy including the terminal ileum [6, 25, 33]. During upper gastrointestinal endoscopy, at least two biopsy specimens should be obtained from the esophagus, stomach, and duodenum, regardless of the presence or absence of upper gastrointestinal symptoms [25]. During both upper and lower gastrointestinal endoscopies including the terminal ileum, specimens must be obtained from endoscopically normal sites [6].

The rate of IBD-U among children is higher than that among adults, and the diagnosis may be changed from UC to CD or from IBD-U to UC or CD during the course of the disease [4, 34]. Furthermore, because endoscopic findings can fluctuate rapidly over a short period, repeated endoscopy examinations are recommended when a discrepancy exists between the endoscopic and histopathologic findings [25].

##### Endoscopic findings of ulcerative colitis

The diagnosis of UC is based on the confirmation of typical chronic inflammation in the colon by lower gastrointestinal endoscopy including the terminal ileum and histopathology, as well as findings that rule out CD or infection. No uniform endoscopic or histopathologic criteria exists for diagnosing UC in children, and many atypical cases that do not fit the diagnostic criteria have been discovered.

The most reliable endoscopic finding of UC is continuous mucosal inflammation extending from the rectum without involvement of the small bowel. Other typical endoscopic findings include erythema, fine granular shadows, fragility, purulent mucus deposits, and ulceration (usually superficial small ulcers) [35]. Inflammation may extend continuous from the rectum to the mouth, terminating at transitional zones or involving the entire colon.

Atypical endoscopic findings associated with UC, include rectal sparing, backwash ileitis, cecal patch, and upper gastrointestinal lesions [6, 36–38]. Albeit endoscopy shows that the a continuous lesion from the rectum, histopathologic findings may reveal localized inflammation without chronic inflammation or abnormal construction of the crypts (short duration of the disease variant) or full-layer inflammation or deep ulceration (V-shaped fissuring ulcers, acute severe colitis) [6, 35]. In such atypical cases, UC should not be ruled out; instead, the diagnosis should be made based on a comprehensive evaluation and supported by clinical symptoms, endoscopic findings (including an evaluation of small intestinal lesions, if necessary), histopathologic findings, and blood test results. However, until the patient presents with characteristic findings during the course of the disease and a final diagnosis is made, the case may be considered IBD-U for convenience during follow-up.

Inflammation associated with UC tends to be more intense distally (anorectal side); conversely, if inflammation is more intense proximally (mouth side), then the diagnosis of UC should be reconsidered, unless it is caused by rectal sparing [6, 39].

*Rectal sparing* Rectal sparing is defined as the absence of inflammation or mild inflammation of the rectum or sigmoid colon despite the fact that lesions on the oral side of the ascending colon are consistent with UC [40]. Rectal sparing is found in 5–30% of pediatric patients with UC (5% [28/533 patients] in the EUROKIDS study) [6, 39]. The frequency of rectal sparing is inversely related to the age at the time of disease onset [39].

*Backwash ileitis*

Backwash ileitis comprises mild inflammation without stenosis, such as nonerosive erythema or edema at the end of the ileum. The entire colon or the ileocecal valve is inflamed. Backwash ileitis is found in 6–20% of all colitis-type UC cases in adults; however, it was also observed in children (10%; 30/296 patients) during the EUROKIDS study [6, 41–44]. The severity of backwash ileitis depends on the degree of inflammation in the right colon [41, 42].

*Cecal patch* Inflammation in the ileocecal area (usually around the appendix), which is a left-sided type of colitis, is called a cecal patch. The cecal patch was observed in approximately 2% of patients (11/578 patients) diagnosed with UC in the EUROKIDS study [39].

*Upper gastrointestinal lesions* Erosions and small ulcers in the stomach (without meandering or linear lesions) are often observed with UC, with a prevalence of 4–8%; furthermore, the EUROKIDS study observed erosions or nonmeandering small ulcers in approximately 4% of patients (11/260 patients) with UC [39, 45, 46]. Nonspecific inflammation of the upper digestive tract and small intestine is considered consistent with the presentation of UC. Additionally, the presence of small erosions in the upper gastrointestinal tract or small intestine does not rule out UC.

#### Endoscopic findings associated with Crohn’s disease

With CD, lesions can occur anywhere in the gastrointestinal tract; however, discontinuous aphthae and linear ulcers are found primarily in the ileum or colon [6]. Typical endoscopic findings of pediatric CD include the following: aphthous ulcers and linear or tortuous ulcers; cobblestone appearance; narrowing of the intestinal tract with prestenotic dilation; anal lesions (anal fissures, abscesses, anal stenosis, anal canal ulcers, inflammation of the hilum); skip stone lesions (skip lesions); and ulcers of the small intestine (jejunum and ileum) [6, 18]. Endoscopic findings not specific to pediatric CD include edema, erythema, fragility, and granular lesions [6, 18].

With colonic CD, the keys to differentiating UC from IBD-U are skip lesions, upper gastrointestinal lesions (especially serpiginous ulcers), a cobblestone appearance, and gastrointestinal stenosis [18]. Aphthous ulcers are typical of CD but are rarely observed with UC, and deep serpiginous ulcers (found anywhere in the gastrointestinal tract) and a cobblestone appearance are characteristic endoscopic findings of CD [6]. Rectal sparing may be observed with UC. However, if histopathologic findings also include rectal sparing, then the diagnosis of CD may be made years later [6, 47]. Although backwash ileitis can be observed with UC, ileitis without inflammation in the cecum or with fissuring ulceration ileitis without cecal inflammation, or ileitis with fistulous ulcers can suggest the possibility of CD [6, 44].

#### Endoscopic findings associated with inflammatory bowel disease unclassified

There are no specific endoscopic findings associated with IBD-U. IBD-U is defined as inflammation primarily of the colon with a definite diagnosis of IBD but without a diagnosis of UC or CD after careful examination [48].

#### Endoscopic severity assessment

During endoscopy, an assessment of severity should be performed in conjunction with the diagnostic evaluation. No specific endoscopic severity assessment exists for children; therefore, the same index used for adults is employed. The main indices are the Mayo Endoscopic Subscore, Ulcerative Colitis Endoscopic Index of Severity, Simple Endoscopic Score for Crohn’s Disease, and Crohn’s Disease Endoscopic Index of Severity [49–52]. No endoscopic severity assessment exists for IBD-U.

*Mayo endoscopic subscore* The Mayo Endoscopic Score is based on mucosal findings and used as an indicator of UC disease activity. A Mayo Endoscopic Subscore of 0 or 1 represents endoscopic mucosal healing, and a decrease of ≥ 1 point is defined as endoscopic improvement (Table 6) (CQ9) [49].

**Table 6:** Mayo endoscopic subscore

Endoscopic findings	Score
No or inactive findings	0
Mild (erythema, decreased vascular translucency, mild fragility)	1
Moderate (marked erythema, loss of vascular translucency, fragility, erosions)	2
Severe (spontaneous bleeding, ulcers)	3

*Simple endoscopic score for Crohn’s disease* The small and large intestines are divided into five regions, and the severity of the disease is determined by evaluating all endoscopic findings in each region (Table 7) [51]. Although the definition of severity has not yet been standardized, clinical studies have defined it as mild (score, 3–6), moderate (score, 7–15), or severe (score, > 15; some clinical studies consider 16 to be severe). The five regions are the ileum (to the extent observable, not including the ileal valve or ileocecal anastomosis), right colon (the ascending colon up to the ileocecal valve, cecum, and hepatic flexure; if localized in the cecum, then this site is scored), transverse colon, left colon (the descending colon to the sigmoid colon junction), and rectum.

**Table 7:** Simple endoscopic score for Crohn’s disease

Factor	Scores			
	0	1	2	3
Ulcer size	None	Aphthous ulcer (φ 0.1–0.5 cm)	Ulcer (φ 0.5–2 cm)	Large ulcer (φ > 2 cm)
Ulcer area	None	< 10%	10–30%	> 30%
Lesion area	None	< 50%	50–75%	> 75%
Stenosis	None	One stenotic site, passage is possible	Multiple ulcers, passage is possible	Passage is not possible

### CQ3: Is histopathology useful for diagnosing pediatric inflammatory bowel disease?

#### Statement

- Distinguishing UC from CD in children is often challenging; however, upper gastrointestinal tract endoscopic and histopathologic findings may be useful.
- A histopathological examination is useful for distinguishing pediatric-specific diseases, such as gastrointestinal allergies and autoimmune diseases.
- A histopathological examination may help differentiate IBD from non-IBD in children.

#### Commentary

The diagnosis of IBD is made based on clinical symptoms, blood test results, endoscopic images, and pathological examination results. Most cases of UC and CD can be differentiated using endoscopic and histopathologic images, which are also useful for excluding diseases from the differential diagnosis.

Histopathological examinations for diagnosing pediatric IBD often do not provide a clear distinction between UC and CD based on initial findings; however, subsequent follow-up and additional histopathological assessments can aid in differentiating the two. Very early-onset IBD, which occurs during infancy, has been described previously [6].

##### Histopathologic characteristics of ulcerative colitis and Crohn’s disease cases

UC exhibits the following characteristics: continuous inflammation with neutrophil infiltration of the colon mucosa; inflammation superficial to the mucous membrane fascia (the mucous membrane fascia is preserved); neutrophil infiltration in the crypts and epithelial cell layer (with crypt abscesses); prominent crypt atrophy and twisting; and plasma cell infiltration in the crypt floor.

CD is characterized by discontinuous inflammatory cell infiltration in the upper and lower gastrointestinal tracts, the presence of nondesmoplastic granulomas, localized distribution of an abnormal arrangement of crypts, and the rare presence of crypt atrophy [53, 54].

##### Histopathologic characteristics of inflammatory bowel disease and noninflammatory bowel disease

In the absence of histopathologic findings characteristic of IBD, non-IBD should be considered when crypt atrophy, crypt torsion, plasma cell infiltration of the crypt base with severe mononuclear cell infiltration, and Paneth cellular transformation on the antral side of the hepatic fold are observed [53, 54].

##### Histopathological examination of the upper gastrointestinal tract

The most common upper lesions observed with IBD are localized neutrophilic infiltrates of the stomach and duodenum. The sensitivity and specificity of the histopathologic examination of upper lesions, including those in adults, for differentiating IBD from non-IBD are 36–41% and 94–97%, respectively, making these lesions an important finding [55–60].

### CQ4: Are small bowel capsule endoscopy, balloon enteroscopy, and magnetic resonance enterography useful for diagnosing pediatric inflammatory bowel disease?

#### Statement

- An imaging evaluation of small bowel lesions is recommended to accurately diagnose pediatric IBD.
- Three types of imaging evaluations of small bowel lesions are useful for children: SBCE, balloon small bowel endoscopy (BAE), and MRE. A comprehensive understanding of each method is necessary. The method of examination should be selected after considering the physique of the child.

#### Commentary

An imaging evaluation of small bowel lesions is recommended to accurately diagnose pediatric IBD. This is especially essential when CD is suspected based on the medical history, or when upper or lower gastrointestinal endoscopic results reveal characteristics that are not typical of UC. This section discusses which tests should be used to evaluate small bowel lesions in children with suspected IBD. If none of these tests can be performed at the involved institution, then the patient should be referred to a facility where these tests can be performed.

Three types of imaging evaluations of small bowel lesions are useful for children: SBCE, BAE, and MRE. However, none should be applied uniformly. The evaluation method should be chosen based on a thorough understanding of its characteristics and tailored to each individual patient. The characteristics of each method are listed in Table 8. An explanation of how small bowel lesions should be evaluated to determine the diagnosis of UC, CD, or IBD-U is provided.

**Table 8:** General characteristics of each method

	Characteristics
SBCE	Offers the least invasiveness if mucosal lesions can be visualized and the capsule can be swallowed High sensitivity for lesion detection, but low specificity Patency capsules are necessary to evaluate small bowel patency (contraindicated for cases involving stenosis) When the capsule cannot be swallowed, it can be placed endoscopically using an endoscopic capsule insertion aid (AdvanCE^®^)^a^
BAE	Inflammation, stenosis, and other lesions can be directly observed, and procedures, such as biopsy and dilatation, can be performed Usually more invasive than SBCE and MRE Requires deep sedation or general anesthesia Risk of radiation exposure when fluoroscopy is used Risk of the inability to reach the lesion site
MRE	Particularly useful for cases of suspected intestinal stenosis and the evaluation of extraintestinal complications Because the intestinal tract must be dilated at the time of imaging, it is necessary to ensure that the prescribed amount of laxative is administered orally or injected through a nasogastric tube Because of the risk of aspiration of laxatives, patients requiring sedation usually cannot be examined

*BAE* balloon small bowel endoscopy, *MRE* magnetic resonance enterography, *SBCE* small bowel capsule endoscopy

^a^Endoscopic implantation using an endoscopic capsule insertion aid (AdvanCE^®^) is covered by the Japanese National Health Insurance for children aged < 15 years who are unable to swallow capsules

##### Ulcerative colitis

The purpose of the small bowel evaluation for suspected UC cases is to rule out CD. Even when the initial diagnosis is UC, a small bowel evaluation often leads to a change in the diagnosis to CD [61–63]. Atypical findings of UC include rectal sparing, backwash ileitis, cecal patch, and upper gastrointestinal lesions (see CQ2). An imaging evaluation of small bowel lesions is required when these findings are observed. SBCE and MRE are preferred because of the invasiveness of BAE; however, no high-quality studies have compared the superiority of these methods. Some studies have indicated the difficulty in distinguishing UC from CD without ileal terminal lesions using MRE [64]. Therefore, diagnosing UC based on MRE results alone is not recommended [65].

##### Crohn’s disease

An imaging evaluation of small bowel lesions is mandatory for suspected CD cases. The characteristics of each method should be used as references when determining which to use in clinical practice. Other characteristics such as the child’s size and age, whether sedation is required, and whether the method can be performed at the examiner’s own facility should also be considered.

*Small bowel capsule endoscopy* In a meta-analysis by Cohen et al., the diagnostic rate of SBCE was 58–72%, which was significantly superior to those of small bowel angiography (0–33%) and ileoscopy (0–61%) [66]. In contrast, although a systematic review that compared SBCE and MRE showed no significant difference in the diagnostic rate with regard to suspected CD (odds ratio, 3.24; 95% confidence interval, 0.14–72.76; *P* = 0.46), SBCE exhibited superior performance when evaluating the proximal small bowel (odds ratio, 2.79; 95% confidence interval, 1.2–6.48; *P* = 0.02) [67]. Because of the risk of capsule retention, small bowel patency should be evaluated using a patency capsule before attempting SBCE. If small bowel patency is ensured, then the risk of capsule retention is < 1% [67]. According to a multicenter study performed in Japan, children with an average age of 9.2 years are usually able to safely swallow a capsule endoscope [68]. However, an endoscopic insertion aid was once used for an 8-month-old child with a weight of 7.9 kg (youngest patient to date) [69, 70]. Iwama et al. reported that endoscopic insertion aids could be used without major adverse events [71].

*Balloon small bowel endoscopy* There have been limited reports of CD alone. One study that included children reported that BAE had a diagnostic rate of 79% (34/43 patients) for suspected CD; however, a study performed in Japan reported a diagnostic rate of 86% (7/8 patients) [72, 73]. SBCE has a high sensitivity for detecting lesions; therefore, BAE may be an advantageous option when SBCE reveals lesions but does not indicate a definitive diagnosis, thus necessitating a careful examination including histopathologic findings.

According to a multi-institutional prospective study in Japan, oral double-balloon small bowel endoscopy was performed for a 3.7-year-old patient with a weight of 12.9 kg (youngest patient to date), and transanal double-balloon small bowel endoscopy was performed for a 1.6-year-old patient with a weight of 10.8 kg (youngest patient to date). The same study also examined the safety of these procedures, and no contingencies required additional procedures, such as IBD scrutiny, during normal observation [74].

*Magnetic resonance enterography* In a systematic review by Yoon et al., the sensitivity and specificity of MRE were 83% and 93%, respectively, which were comparable to its diagnostic rates for adults [75]. However, MRE is more likely than SBCE to underestimate the disease severity and is less sensitive when detecting lesions in the proximal small intestine. MRE requires reliable oral administration of a prescribed dose of laxatives at a set time. This limits the examination, especially for children who require sedation at the time of imaging; however, some facilities have performed MRE without sedation for children 4–7 years of age with the support of a child life specialist, and others have performed MRE under general anesthesia for children 1 month to 9 years of age [76, 77]. However, the use of MRE alone for diagnosing CD is not recommended.

##### Inflammatory bowel disease unclassified

IBD-U should be considered when inflammation is observed in the colon, the diagnosis of IBD is certain, and typical UC and CD cannot be diagnosed based on upper or lower gastrointestinal endoscopic or histopathologic findings. Therefore, the purpose of the small bowel evaluation for such cases, as with UC cases, is to rule out CD. Several studies have reported that the diagnosis of IBD-U was changed to CD after a small bowel evaluation was performed [61–63]. However, within the scope of our search, we were unable to find any studies that evaluated whether a small bowel examination can be used to diagnose IBD-U.

### CQ5: Is abdominal ultrasonography useful for diagnosing pediatric inflammatory bowel disease?

#### Statement

- The diagnosis of UC using abdominal ultrasonography (AUS) alone is not recommended as no evidence supports its efficacy.
- Although the diagnostic performance of AUS is comparable to that of other imaging modalities used for CD, no studies have demonstrated or compared the specificity of AUS for diagnosing CD and other gastrointestinal diseases; therefore, AUS alone should not be used to diagnose CD.
- AUS alone is not recommended for diagnosing IBD-U.

#### Commentary

AUS includes bowel ultrasonography, which focuses on the intestinal tract only. However, bowel ultrasonography is described here as AUS because it is used to address findings outside the intestinal tract.

##### Ulcerative colitis

One systematic review of the diagnosis of UC and ultrasonography included adult and pediatric cases. Of the 6769 articles that met the eligibility criteria, 50 were selected and analyzed. According to the review, in most studies, bowel wall thickening and increased color Doppler blood flow signal were used to assess disease activity and lesion distribution in UC, and AUS was able to accurately assess the affected area, severity, and response to treatment [78]. However, intestinal wall thickening with UC is also seen with CD, acute infectious colitis, ischemic colitis, radiation colitis, diverticular disease, and malignant tumors, and no study has demonstrated that AUS alone can differentiate UC from other digestive diseases. Additionally, when UC is mild, it may not be ruled out, even in the absence of abnormal AUS findings, thus making it difficult to diagnose UC with AUS alone [78]. The use of AUS to diagnose pediatric UC has not been mentioned in the ESPGHAN revised Porto criteria [6] or ECCO guidelines [21, 24]. Consequently, it is difficult to diagnose UC using AUS alone. However, AUS can be used to evaluate the localization of UC lesions and the effects of treatment on the entire colon by assessing inflammation according to the colon wall thickness and Doppler blood flow after UC is diagnosed based on the lower gastrointestinal endoscopic and histopathologic results, as described previously [79–81]. Furthermore, AUC is also useful for evaluating extraintestinal complications [82].

##### Crohn’s disease

According to the revised Porto criteria and ECCO guidelines, MRE and SBCE are imaging modalities that can be used to diagnose CD or differentiate CD from UC; however, the efficacy of AUS alone for diagnosing CD were not indicated in these guidelines [6, 21, 24]. Several systematic reviews of AUS for CD have been published. Kopylov et al. compared the diagnostic performance of AUS for CD with that of MRE and SBCE and summarized 13 studies that compared CD diagnosed with MRE and AUS using ultrasound contrast agents [67, 83]. They concluded that active small bowel lesions were observed with CD before and after the diagnosis was made using all three methods for adults and children, and that no significant difference exists in the accuracy of these methods [67]. Lee et al. also summarized 11 studies that compared the ability of AUS and MRE to detect active lesions with CD and found no difference in their diagnostic performance [83]. Chiorean et al. performed a systematic review of AUS images of pediatric CD and found that its diagnostic sensitivity and specificity ranged from 75–94% and 67–100%, respectively; furthermore, AUS exhibited high sensitivity (90–95%) when detecting terminal ileal lesions but decreased sensitivity in the proximal small intestine (75%) and large intestine (82%). The AUS images of CD showed discontinuous asymmetric bowel wall thickening with preserved laminar structure during the early stage of the disease, whereas the laminar structure of the thickened bowel completely disappeared in the presence of advanced disease, resulting in a hypoechoic image. Additionally, the fibrofatty proliferation of the mesentery causes the lesion to appear isolated within the fatty tissue (creeping fat sign), and twisting of the intestinal tract is depicted because of mesenteric tearing. The Doppler blood flow signal in the thickened intestinal wall observed with CD is increased in proportion to disease activity and is used to evaluate the response to treatment [84]. However, numerous review articles have noted that the images obtained with AUS are similar to those of other gastrointestinal diseases, making it difficult to differentiate CD from these diseases using AUS alone.

##### Inflammatory bowel disease unclassified

The usefulness of AUS for diagnosing IBD-U is not discussed in the revised Porto criteria or ECCO guidelines [6, 21, 24]. Diagnosing IBD-U using AUS alone is extremely difficult as no validated systematic reviews have been performed.

### CQ6: Are biomarkers useful for diagnosing pediatric inflammatory bowel disease?

#### Statement

- Biomarkers can help determine whether to perform gastrointestinal endoscopy to diagnose pediatric IBD.
- Fecal calprotectin is useful for differentiating IBD from functional gastrointestinal disorders (e.g., irritable bowel syndrome).
- C-reactive protein (CRP), hemoglobin precipitation, proteinase 3 ANCA (PR3-ANCA), and anti-integrin αVβ6 antibody are blood biomarkers.
- Fecal calprotectin and fecal occult blood are fecal biomarkers.
- Prostaglandin E-major urinary metabolite is a urine biomarker.

#### Commentary

Gastrointestinal endoscopy and histopathology are required to diagnose IBD in children and adults. However, because children often require sedation or anesthesia before endoscopy can be performed, biomarkers are useful aids that can help determine whether endoscopy should be performed to diagnose IBD. Blood, stool, and urine biomarkers have been reported. In addition to diagnostic biomarkers, biomarkers of disease activity (CRP, fecal calprotectin, leucine-rich α2-glycoprotein (LRG), prostaglandin E-major urinary metabolite (PGE-MUM)) have been reported. This section focuses on biomarkers that support the diagnosis of disease.

##### Blood biomarkers

*CRP and erythrocyte sedimentation rate*

CRP and hemoglobin sedimentation, which are measured in routine medical practice, are increased in the presence of pediatric IBD. However, compared to patients with CD, a larger proportion of patients with UC have normal inflammatory biomarkers, including CRP and hemoglobin sedimentation (UC 34% vs. CD 15%) [85]. With moderate to severe UC, because some studies have reported that the normal CRP (≤ 0.8 mg/dL) and hemoglobin sedimentation (≤ 20 mm/h) rates are 40% and 18.7%, respectively, IBD cannot be ruled out even if these rates are normal [86].

*Proteinase 3 ANCA*

Although it is not covered by insurance in Japan, PR3-ANCA is a useful biomarker. PR3-ANCA is an autoantibody against proteinase 3 in neutrophil cytoplasm. In the presence of pediatric UC, the PR3-ANCA level is higher than that of patients with CD and healthy controls, with a cutoff value of 0.8 U/mL, sensitivity of 64.9%, and specificity of 83.1%; furthermore, it is more useful than myeloperoxidase ANCA [87]. PR3-ANCA has been reported to be higher in pediatric UC compared to CD and healthy controls, its diagnostic accuracy is modest, and it is not commonly used in clinical practice. Therefore, while its presence has been noted in some studies, further validation is required before recommending its routine use [88, 89].

*Anti-integrin αVβ6 antibody*

A study performed in Japan found increased anti-integrin αVβ6 antibody levels in adults with UC [16]. Regarding pediatric IBD, 94.7% of UC cases, 32.6% of CD cases, and 20% of IBD-U cases were positive for anti-integrin αVβ6 antibody [17]. In particular, 13 of 15 patients with a diagnosis of UC turned CD were positive for anti-integrin αVβ6 antibody. Because positive anti-integrin αVβ6 antibody levels are observed with IBD-U with UC-like features, anti-integrin αVβ6 antibody may be useful for classifying the disease type and is expected to be applied clinically in the future.

*Leucine-rich alpha 2 glycoprotein*

Leucine-rich α2 glycoprotein is correlated with UC and CD disease activity in adults. Additionally, it is used as a biomarker of disease activity and can help determine the active stage of disease. However, no reports of leucine-rich α2 glycoprotein for diagnosing pediatric IBD have been published.

##### Fecal biomarkers

*Calprotectin in stools*

Calprotectin is a calcium-binding protein that belongs to the S100 protein family, and its release in stools (fecal calprotectin) reflects inflammation of the intestinal tract [90]. Since December 2021, enzyme-linked immunosorbent assays that detect fecal calprotectin have been available for patients without infectious enteritis who do not have grossly bloody stools but have symptoms, such as diarrhea, abdominal pain, or weight loss persisting for > 3 months. However, fecal calprotectin can be measured using an adjunctive test for IBD that is covered by insurance. For newly diagnosed pediatric IBD cases, no difference was noted in the fecal calprotectin levels observed with UC, CD, and IBD-U; however, these levels were significantly increased compared to those of controls [91, 92]. Caution is necessary when interpreting fecal calprotectin levels as they are elevated in the presence of diseases requiring IBD as a differential diagnosis, including gastrointestinal infections and juvenile polyps, and because healthy children younger than 4 years may have increased fecal calprotectin levels [93, 94]. Therefore, for children with chronic diarrhea and abdominal pain, the fecal calprotectin test is useful for differentiating functional gastrointestinal disorders, such as irritable bowel syndrome, before gastrointestinal endoscopy; therefore, its use is recommended.

Fluorescence enzyme immunoassays, enzyme-linked immunosorbent assays, latex agglutination tests, immunochromatography, and gold colloid agglutination tests can be used to measure fecal calprotectin. The amount of specimen and time required to obtain results differ depending on the method, and thus confirmation is necessary.

*Fecal occult blood test*

Fecal occult blood test is an immunological test that specifically evaluates human hemoglobin and is useful for diagnosing pediatric IBD [92, 95]. When diagnosing pediatric IBD, the sensitivity and specificity were 95.1% and 96.6%, respectively, when the cutoff value was 87 ng/mL [92].

##### Urine biomarkers

*Prostaglandin E-major urinary metabolite*

Prostaglandin E2 (PGE2) is a major mediator involved in the promotion and suppression of inflammation, and mucosal inflammation results in increased PGE2 blood levels [96–98]. However, PGE2 released in the blood is rapidly metabolized and has a short half-life, thus adding difficulty to its measurement in the blood. Metabolized PGE2 is excreted in urine, and its major metabolite, prostaglandin E-major urinary metabolite, is stable; therefore, its usefulness as a biomarker of UC activity has been reported [99–101]. Prostaglandin E-major urinary metabolite levels observed with pediatric UC were significantly higher than those of controls, such as functional gastrointestinal disease [100, 102]. Urine can be collected noninvasively and may be useful for diagnosing pediatric IBD [103]. As of January 2024, urine sample measurements performed in clinical practice were not covered by insurance; however, measurements can be performed by a clinical laboratory self-funded.

### CQ7: What are the causes of growth restriction with pediatric inflammatory bowel disease?

#### Statement

- Stunted growth and delayed secondary sexual characteristics are common and important complications of pediatric IBD; therefore, determining their presence using growth curves and the Tanner classification is important.
- Patients with disrupted development present with stunted growth, decreased peak adult height, weight loss, decreased bone mineral density, and delayed secondary sexual characteristics.
- The causes of stunted growth are multifactorial. Factors associated with the underlying disease include decreased food intake because of gastrointestinal symptoms, such as abdominal pain and diarrhea; malabsorption and nutrient leakage caused by intestinal mucosal damage; and increased energy requirements attributable to fever and inflammation. Growth is also disrupted by cytokines and other effects of chronic inflammation. Factors other than the primary disease include prolonged systemic administration of steroids, which can cause stunted growth.
- When stunted growth or delayed secondary sexual characteristics are observed, the patient should be referred to a pediatric endocrinologist for consultation, and the treatment plan, including steroids, should be evaluated.

#### Commentary

##### Delayed growth and development and decreased peak adult height

Stunted growth has been recognized as an important complication of pediatric IBD [104]. Stunted growth includes delayed growth and development, decreased peak adult height, weight loss, decreased bone density, and delayed secondary sexual characteristics. The incidence of stunted growth at the time of diagnosis has been reported to be 15–40% with pediatric CD and 3–10% with pediatric UC [105]. According to a prospective study of pediatric IBD symptoms performed over 2 years in England, the weights of 66% of patients with CD and 33% of patients with UC were below the 10th percentile. Similarly, the heights of 32% of patients with CD and 13% of patients with UC were below the 10th percentile. Jejunal disease was similarly associated with decreased height and weight at the time of diagnosis [106]. In Japan, stunted growth or growth restriction was observed in 7.6% (6/79) of patients with CD and 7.8% (10/128) of patients with UC [3].

Rinawi et al. conducted a retrospective study of peak adult height that included 436 pediatric patients with IBD and found that boys had a significantly lower peak adult height than age-matched controls, and the difference was approximately 1 cm. Girls did not have a significantly different peak adult height than age-matched controls. A younger age at the time of diagnosis was independently associated with a lower peak adult height [107]. Furthermore, adolescent patients with IBD continue to grow taller after the expected time of growth plate closure, suggesting that these patients approach the adult height predicted based on the median heights of their parents [108]. Gupta et al. conducted a retrospective observational study and found that 81% of patients with CD and 75% of patients with UC gained at least 1 cm in height beyond the age at which growth plate closure usually occurs (15 years for female patients and 17 years for male patients) [108]. The median peak adult height was greater for male patients with UC than for male patients with CD; however, no difference was observed in female patients. The authors suggested that although skeletal maturation is delayed with pediatric IBD, it may be possible for patients to grow for a longer period of time when remission is achieved [108].

If stunted growth is suspected, then the following steps should be performed: examine the growth curve and evaluate the delayed growth rate; evaluate the family history and history of small-for-gestational-age status; and examine the bone age, insulin-like growth factor-1 level, thyroid function, and gonadal function [109–111]. If abnormal values are observed, or if it is difficult to diagnose stunted growth, then a pediatric endocrinologist should be consulted (Table 9). The classification of short stature is shown in Table 10. Although IBD is also a risk factor for short stature, it is important to distinguish IBD from other conditions. Moreover, in clinical practice, evaluation of the epiphyseal line using radiography is also a simple and accessible method for assessing bone age and growth potential. This approach can help determine the degree of growth delay and support decisions regarding referral to a pediatric endocrinologist.

**Table 9:** Assessment of suspected stunted growth

	Endpoints
1. Growth curve	Record the growth curve Check for short stature (SD, ≤ − 2.0) and a reduced growth rate (growth rate SD, ≤ − 1.5)
2. Medical history and family history	Evaluate the patient’s history (including small-for-gestational-age status), general condition, chromosomal abnormalities, signs of malformations, and limb balance Evaluate the family history and heights of the parents
3. Blood and bone age tests	Blood test: evaluate anemia, liver function, kidney function, inflammation, thyroid function, gonadal function, bone system disease, and adrenocortical function Bone age test: perform an X-ray examination of the left hand to evaluate the radius, ulna, and phalanges of the left hand, including the epiphyseal cartilage and carpal bones
4. Growth hormone challenge	Evaluate the growth hormone and sex hormone secretion capacities (pediatric endocrinologist)

*SD* standard deviation

**Table 10:** Conditions leading to short stature

Endocrine abnormalities	Abnormal growth hormone secretion
	Abnormal thyroid function
	Excess glucocorticoids
Other causes	Spontaneous
	Short stature associated with small-for-gestational-age
	Congenital anomalies
	Bone system disorders
	Calcium, phosphorus, and vitamin D abnormalities
	Congenital metabolic abnormalities
	Emotional deprivation syndrome
	Chronic diseases (renal, cardiac, and inflammatory diseases, IBD)
	Malnutrition
	Drug use (e.g., steroids)
	Radiotherapy

*IBD* inflammatory bowel disease

##### Bone density

Metabolic bone disease is commonly observed with pediatric IBD, and decreased bone mass is observed at the time of diagnosis [112]. Although an ongoing debate exists on whether decreased bone mineral density in pediatric IBD is associated with an increased fracture risk [113], decreased bone mass during childhood and adolescence may have long-term consequences. Screening performed using dual-energy X-ray absorptiometry is recommended, and follow-up intervals should be determined according to the degree of abnormality [113].

##### Weight loss

A large cohort study of weight loss performed in North America found that up to 24% of patients with CD and 9% of patients with UC had a body mass index below the 5th percentile at the time of diagnosis [114]. Studies performed in the United Kingdom [106] and Japan [3] found similar results regarding weight loss; therefore, evaluating any deviations in the growth curve is essential in clinical practice [115].

##### Delayed secondary sexual characteristics

Children with IBD, especially those with CD, commonly have delayed secondary sexual characteristics. Gupta et al. used the National Health and Nutrition Examination Survey to compare 34 female patients with CD and reported that the median age at menarche was 12.0 years for healthy controls but 13.9 years for patients with CD [116]. Studies on delayed secondary sexual characteristics among boys is limited, highlighting the need for further research.

##### Steroids

Steroids are important to the treatment and remission of pediatric IBD. However, their prolonged use has been associated with decreased bone mineral density [112]. Some studies have suggested that steroid-induced stunted growth is mediated by insulin-like growth factor, which acts on cartilage cells of the growth plate in a manner that decreases skeletal metabolism [108]. Decreased growth rates during steroid therapy are well-known, and cessation of height growth during treatment is common. A study performed in Japan found that steroid therapy for pediatric IBD is associated with short stature [117]. Therefore, steroid therapy should be minimized during adolescence, which is when rapid increases in height are often observed.

##### Multifactorial pathogenesis of stunted growth

**Fig. 3:**
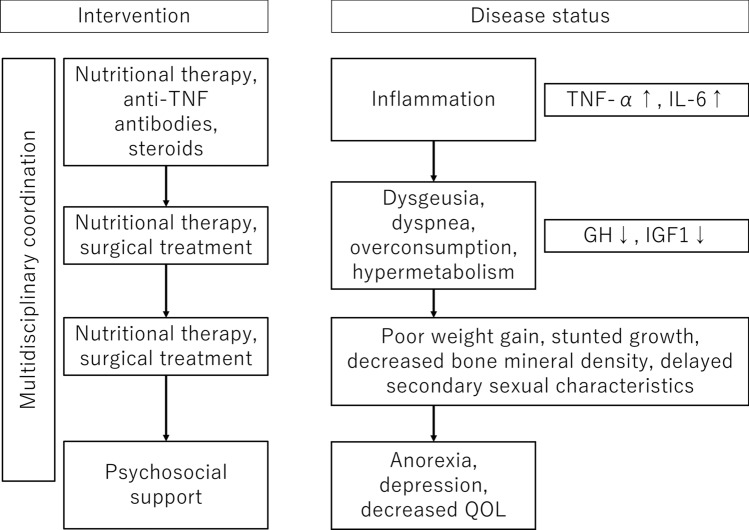
Schematic diagram of clinical manifestations, pathological mechanisms, and therapeutic interventions associated with pediatric inflammatory bowel disease (IBD)-related stunted growth. Evidence for all pathways has not been fully established. Adapted from [10], with partial modification. *GH* growth hormone, *IGF* insulin-like growth factor, *IL* interleukin, *QOL* quality of life, *TNF* tumor necrosis factor

Similar to the multifactorial pathogenesis of IBD, the process that causes stunted growth is also multifactorial. Factors associated with the underlying disease include decreased food intake as a result of gastrointestinal lesions, diminished appetite, abdominal pain, and diarrhea, malabsorption and nutrient leakage caused by intestinal mucosa damage, and increased energy requirements attributable to fever and inflammation. Additionally, when cytokine (tumor necrosis factor-α) production increases as a result of chronic inflammation, growth is stunted by multiple pathways, including the inhibition of insulin-like growth factor-1 caused by increased interleukin-6, inhibition of growth hormone receptors, and decreased leptin (Fig. 3) [115]. Prolonged systemic administration of steroids can also result in stunted growth [117]. Therefore, an assessment of stunted growth should be performed at the beginning of pediatric IBD treatment, efforts should be made to minimize steroid use during treatment, and growth should be adequately monitored and managed throughout the course of the disease.

### CQ8: Which classification is recommended for pediatric inflammatory bowel disease?

#### Statement

- The Paris Classification is recommended to determine the type of pediatric IBD (UC or CD).

#### Commentary

The Montreal Classification [118] was proposed in 2005 to classify the disease type and promote IBD treatment and research. Subsequently, the Montreal Classification was deemed inadequate for classifying clinical features, such as the extent of disease, pathological changes over time, and growth disturbances of patients with childhood-onset IBD; therefore, the Paris Classification [18] was proposed in 2009 to define these new features. The Paris Classification provides more detailed descriptions of items that should be considered during the treatment of pediatric IBD. Although the IBD practice guidelines for adults were determined according to the Montreal Classification [119], the ESPGHAN practice guidelines for UC and CD in children [23, 120] were determined according to the Paris Classification.

##### Ulcerative colitis

The Paris Classification of UC is shown in Table 11 and includes the following: E1, proctitis (limited to the rectum); E2, left colitis (anorectal to the splenic flexure); E3, extensive colitis (anorectal to the hepatic flexure); and E4, total colitis (oral to the hepatic flexure). The Montreal Classification only includes classes E1, E2, and E3. The total colitis type (E4) has been observed more commonly with childhood-onset UC cases in Japan [121] and is important in diagnosing the extent of the disease. Furthermore, during the evaluation based on the Pediatric Ulcerative Colitis Activity Index [122], the history of severe disease is specific to pediatric cases.

**Table 11:** Paris classification of UC

Involvement	E1	Ulcerative proctitis
	E2	Left colitis (anorectal side above the splenic flexure)
	E3	Extensive lesions (anorectal side above the hepatic curvature)
	E4	Total colitis (oral side of the hepatic curvature)
Severity	S0	No history of severe disease
	S1	History of severe disease

Severe disease: Pediatric Ulcerative Colitis Activity Index ages ≥ 65 years

*UC* ulcerative colitis

##### Crohn’s disease

The Paris Classification for CD is shown in Table 12. Montreal Classification A is assigned when the diagnosis is made when the patient is 16 years or younger; however, the Paris Classification divides class A into A1a (0–9 years) and A1b (10–16 years). This division is attributable to differences in the location and extent of lesions according to the age of onset as well as differences in disease susceptibility genes, which are considered important to the phenotypic classification. The Montreal Classification divides lesions into the following classes: L1, small bowel type; L2, large bowel type; L3, small bowel colorectal type; and L4, isolated upper lesions according to the Montreal Classification. L4 lesions are divided into oral and anal lesions in relation to the Treitz ligament. Disease activity of the jejunum is considered to be involved in stunted growth [106], and the contents of the Paris Classification are more detailed than those of the Montreal Classification for the small intestine. The Paris Classification includes the following: B1, nonstricturing and nonpenetrating; B2, stricturing; B3, penetrating; B2B3, both penetrating and stricturing disease, either at the same or different times; and P, perianal disease modifier. In addition to these classes, there is an item that is used to evaluate the presence of stunted growth. Therefore, the Paris Classification is considered more specific to pediatric cases than the Montreal Classification in terms of CD.

**Table 12:** Paris classification of CD

Age at the time of diagnosis	A1a	0–9 years
	A1b	10–16 years
	A3	17–40 years
	A3	≥ 41 years
Extent of involvement	L1	Localized lesions in the ileum with or without the cecum in the anorectal one-third
	L2	Colorectal lesions
	L3	Iliac and colorectal lesions
	L4a	Upper lesion on the oral side above the Treitz ligament
	L4b	Upper lesion anorectally above the Treitz ligament and more orally than the terminal one-third of the ileum
Disease state	B1	Inflammatory lesions without stenosis or perforation
	B2	Stenotic lesions
	B3	Perforating lesions
	B2B3	Stenotic/perforating lesions
	P	With anal lesions (only with hemorrhoids, anal canal ulcers, or abscesses)
Growth	G0	Stunted growth not present
	G1	Stunted growth present

*CD* Crohn’s disease

##### Inflammatory bowel disease unclassified

We could not find any evidence-based classification of IBD-U.

### CQ9: How is the severity of pediatric inflammatory bowel disease classified?

#### Statement

- The Pediatric Ulcerative Colitis Activity Index is recommended for measuring pediatric UC disease activity.
- The Pediatric Crohn’s Disease Activity Index and weighted Pediatric Crohn’s Disease Activity Index are recommended for measuring pediatric CD disease activity.
- Although there have been no reports of a severity classification specific to IBD-U, if the disease type is similar to that of UC or CD, then the respective severity class may be adopted.

#### Commentary

##### Ulcerative colitis

Compared to adult UC cases, pediatric UC cases are characterized not only by more extensive lesions in the colon but also by greater severity [123]. Endoscopy is more invasive for children than for adults because of the need for anesthesia; therefore, the Pediatric Ulcerative Colitis Activity Index, which is a noninvasive index that can accurately measure disease activity associated with pediatric UC, was created by Turner et al. [122]. Their study included 36 pediatric IBD specialists in North America and 11 endpoints that were extracted from existing UC indices and weighted based on a cohort of patients with pediatric UC. Six items, abdominal pain, rectal bleeding, fecal characteristics, number of bowel movements per day, nighttime bowel movements (nocturnal arousal), and activity level, were divided into two to four levels with scores ranging from 0 to 30. Based on the total score of these six items, the disease was classified as one of the following three categories: mild (Pediatric Ulcerative Colitis Activity Index = 10–30); moderate (Pediatric Ulcerative Colitis Activity Index = 35–60); and severe (Pediatric Ulcerative Colitis Activity Index = 65–85) (Table 13). A validation study found good correlations between the Pediatric Ulcerative Colitis Activity Index and endoscopic findings, the Mayo Score (Table 14), and the revised version of the Truelove-Witts Index [122]. A recent validation study of the Pediatric Ulcerative Colitis Activity Index in daily practice that included 2503 patients with pediatric UC showed a good correlation with the Physician Global Assessment, indicating that the Pediatric Ulcerative Colitis Activity Index is a highly reliable index [124]. The severity classification of the Research Group on Intractable Inflammatory Bowel Disease (hereafter referred to as the Research Group) is used in Japan. Although it is not specifically for pediatric IBD, it is frequently used to assess adult UC cases and has been adopted for specific diseases [1].

**Table 13:** Pediatric ulcerative colitis activity index

		Score
Stomach pain	None	0
	Bearable	5
	Unbearable	10
Rectal bleeding	None	0
	A small amount of bleeding is observed with < 50% of bowel movements	10
	A small amount of bleeding is observed with almost every bowel movement	20
	Heavy bleeding (> 50% of stool volume)	30
Stool properties	Shaped	0
	Partially shaped	5
	Completely shapeless	10
Bowel movements per day, no	0–2	0
	3–5	5
	6–8	10
	≥ 9	15
Nighttime bowel movements	None	0
	Yes	10
Activity level	No interference with activities	0
	Some interference with activity	5
	Severe interference with activity	10

Maximum score = 85. Remission = < 10; mild = 10–30; moderate = 35–60; and severe = 65–85

**Table 14:** Mayo score

Scores are based on findings over the course of 3 days		Score
Bowel movements, no	Once per day (or normal number)	0
	More than 1–2 times/day	1
	More than 3–4 times/day	2
	More than 5 times/day	3
Bloody stools	No bloody stools	0
	Slight presence of blood in less than half of all bowel movements	1
	Blood is clearly present in most bowel movements	2
	Bowel movement comprises a large quantity of blood	3
Mucosal findings	Normal or inactive findings	0
	Mild (erythema, decreased vascular translucency, mild fragility)	1
	Moderate (marked erythema, loss of vascular translucency, fragility, erosions)	2
	Severe (spontaneous bleeding, ulcers)	3
PGA	Normal	0
	Mild disease	1
	Moderate disease	2
	Severe disease	3

*PGA* Physician Global Assessment

Maximum score = 12. Remission = ≤ 2; mild = 3–5; moderate = 6–10; and severe = 11–12

##### Crohn’s disease

Compared to adult-onset CD, pediatric CD is characterized by more extensive and severe lesions and stunted growth at the time of diagnosis [123]. Therefore, the Pediatric Crohn’s Disease Activity Index was developed by 30 pediatric gastroenterologists in North America with pediatric IBD expertise (Table 15) [125]. A high interobserver agreement rate between two pediatric gastroenterologists and a favorable correlation with the Physician Global Assessment were reported [125], and the results of a validation study showed that the Pediatric Crohn’s Disease Activity Index is a reliable index [125].

**Table 15:** Pediatric Crohn’s disease activity index

				Score
Medical history (1-week recall) Stomach pain	None			0
	Mild: brief, ADLs unimpeded			5
	Moderate/severe: restricted activity and nocturnal symptoms for extended periods and multiple days			10
Patient behavior/general condition	Good, ADLs unimpeded			0
	Occasional limitations and substandard maintenance of age-appropriate activities			5
	Frequent limitations of ADLs, very impaired			10
Frequency and nature of bowel movements	0–1 watery stools, no blood in stools			0
	2 or more semi-organic stools or 0–5 liquid stools with a small amount of blood			5
	Obviously bloody stools or > 6 watery stools or diarrhea at night			10
Blood tests				
Hematocrit value	Boys and girls 10 years or younger		Boys 11–14 years	
	≥ 33%		≥ 35%	0
	28–32%		30–34%	2.5
	< 28%		< 30%	5
	Girls 11–19 years		Girls 15–19 years	
	≥ 34%		≥ 37%	0
	30–33%		32–36%	2.5
	< 29%		< 32%	5
Erythrocyte sedimentation rate (mm/h)	< 20			0
	20–50			2.5
	> 50			5
Albumin (g/dL)	≥ 3.5			0
	3.1–3.4			5
	≤ 3.0			10
Examination findings				
Shifts in body weight	Weight gain or intentional weight changes/weight loss			0
	Unintentional weight changes, 1–9% decrease in weight			5
	≥ 10% decrease in body weight			10
Height	At the time of diagnosis	Observation time		
	< 1 change	Growth rate SD ≥ − 1		0
	> 1 change but < 2 changes	Growth rate SD <− 1 but > − 2		5
	≥ 2 changes	Growth rate SD ≤ − 2		10
Abdomen	No tenderness or ulceration			0
	Tenderness present or a mass without tenderness			5
	Tenderness present, muscular defense, or obvious mass			10
Anal lesions	None/only asymptomatic cutaneous edema			0
	1–2 painless fistulas with poor drainage and no tenderness			5
	Active fistula with drainage, tenderness, or abscess			10
Extraintestinal complications (fever > 38.5 °C for > 3 days during the past week, arthritis, iritis, erythema nodosum, pyoderma gangrenosum)	None			0
	1			5
	≥ 2			10

Maximum score = 100. Remission = < 10; mild = 10–30; moderate = 30–70; and severe = 70–100

The changes (decreases) in the 3, 5, 10, 25, 50, 75, 90, 95, and 97 percentiles of the growth curve at the time of diagnosis were evaluated and compared with those before disease onset. If a measurement that was in the 50th percentile before symptom onset was in the 10th percentile at the time of diagnosis, then the change crossed the 25th percentile and was considered a change of ≥ 1 but < 2, resulting in a score of 5 points

*ADLs* activities of daily living

The Pediatric Crohn’s Disease Activity Index is not versatile in clinical practice because of the inclusion of laboratory values, anorectal examination findings, and growth rate, and because it is not suitable for short-term evaluations of the treatment response (weighted Pediatric Crohn’s Disease Activity Index) (Table 16) [126].

**Table 16:** Weighted pediatric Crohn’s disease activity index

Item		Score
Stomach pain	None	0
	Mild: brief and does not limit activity	10
	Moderate/severe: prolonged, daily, activity-limiting, or occurs after sleep Good condition, no activity restrictions	20
Patient behavior/general condition	Good condition, behavior unrestricted	0
	Age-appropriate behavior may be more restricted than usual	10
	Poor condition, often with behavioral restrictions	20
Stools (per day)	0–1, no blood	0
	≤ 2 soft stools with a small amount of blood or 2–5 watery stools	7.5
	Obvious bleeding or > 6 watery stools or diarrhea after sleeping	15
Erythrocyte sedimentation rate (mm/h)	< 20	0
	20–50	7.5
	> 50	15
Albumin (g/dL)	≥ 3.5	0
	3.1–3.4	10
	≤ 3.0	20
Shifts in body weight	Weight gain or intentional weight changes/weight loss	0
	Unintentional weight changes, 1–9% decrease in weight	5
	≥ 10% decrease in body weight	10
Anal lesions	None/only asymptomatic cutaneous edema	0
	1–2 painless fistulas with poor drainage and no tenderness	7.5
	Active fistula with drainage, tenderness, or abscess	15
Extraintestinal complications (fever > 38.5 °C for > 3 days during the past week, arthritis, iritis, erythema nodosum, pyoderma gangrenosum)	None	0
	At least 1 extraintestinal complication	10

Maximum score = 125. Remission = ≤ 10; mild = 12.5–40; moderate = 40–57.5; and severe = 57.5–125

##### Inflammatory bowel disease unclassified

No studies have focused on activity indices specific to pediatric IBD-U, nor are there any recommended indices for this condition. Because IBD-U in children is often rediagnosed as UC or CD later in life [9, 10], the use of the Pediatric Ulcerative Colitis Activity Index or Pediatric Crohn’s Disease Activity Index during the IBD-U diagnostic period to allow for later review is ideal.

## Abbreviations

IBD: Inflammatory bowel disease
UC: Ulcerative colitis
CD: Crohn’s disease
IBD-U: Inflammatory bowel disease unclassified
